# 2285. Using Genomic Sequencing to Describe SARS-CoV-2 Transmission Dynamics in U.S. Major League Soccer Clubs

**DOI:** 10.1093/ofid/ofad500.1907

**Published:** 2023-11-27

**Authors:** Joseline Velasquez-Reyes, Ludy R Carmola, Jacquelyn Turcinovic, Isaac Schneider, Margot Putukian, Kyle Sherry, Natalie Akula, Owen Rischmann, Holly Silvers-Granelli, Bradley A Connor, Kristina Angelo, Phyllis E Kozarsky, Michael Libman, Ralph Huits, Davidson H Hamer, Daniel Bourque, Jessica K Fairley, Anne Piantadosi, John Connor

**Affiliations:** Boston University School of Medicine, Boston, Massachusetts; Emory University School of Medicine, Atlanta, Georgia; Boston University, Boston, Massachusetts; Emory University Rollins School of Public Health, Atlanta, Georgia; Major League Soccer, New York, New York; Major League Soccer, New York, New York; Major League Soccer, New York, New York; Major League Soccer, New York, New York; Major League Soccer, New York, New York; New York Center for Travel and Tropical Medicine, New York, New York; Centers for Disease Control and Prevention, Atlanta, Georgia; Emory University School of Medicine, Atlanta, Georgia; McGill University; IRCCS Ospedale Sacro Cuore Don Calabria, Verona, Veneto, Italy; Boston University School of Public Health, MA; Boston Medical Center / Boston University School of Medicine, Boston, Massachusetts; Emory University, Division of Infectious Diseases, Atlanta, Georgia; Emory University School of Medicine, Atlanta, Georgia; Boston University, Boston, Massachusetts

## Abstract

**Background:**

In 2020–2022, U.S. Major League Soccer (MLS) used SARS-CoV-2 mitigation protocols that included masking, social distancing, avoiding contact with others outside of training and games, testing, quarantine, and isolation. In addition to isolation for those who tested positive, a SARS-CoV-2 genomic sequencing strategy was developed to identify whether infections were associated with intra-league transmission.

**Methods:**

Nasopharyngeal swabs were collected by MLS club medical staff from players and staff from February 2021 through September 2022. During this time, surveillance testing changed from daily to weekly to testing only symptomatic players and staff.

SARS-CoV-2 positive samples were analyzed using amplicon-based whole genome sequencing. A phylogenetic tree was constructed using Nextstrain to visualize genomes from infected players and staff in the context of genomes available on GISAID circulating in the United States during corresponding time periods. To identify transmission links, all genomes were compared in a pairwise fashion. Genomes that were 0–2 nucleotides different were considered part of direct transmission clusters.

**Results:**

Of 250 samples that were rtPCR positive for SARS-CoV-2, 215 (86%) were sequenced. Phylogenetic analysis showed a broad diversity of lineages, including some predominant overseas (e.g., AY.98.1). Pairwise comparison revealed SARS-CoV-2 sequences from 7 individuals appeared to be linked. These individuals were on 2 different MLS teams; there was an off-field exposure (dinner). Five (71%) had identical SARS-CoV-2 sequences; 2 had sequences that were 1 nucleotide different at the consensus level. Subconsensus genome analysis revealed that differences of 1 nucleotide were due to subconsensus mutations fluctuating below and above 50%, suggesting all 7 individuals were part of a transmission cluster. Phylogenetic analysis revealed that the sequences from the cluster were distinct from reference sequences and were likely a unique transmission chain.

**Conclusion:**

SARS-CoV-2 transmission may occur within, and even between, professional sports teams. Pairwise comparison of individual genomes can provide improved granularity to identify clusters and understand SARS-CoV-2 transmission.

**Disclosures:**

**Davidson H. Hamer, MD**, Kephera Diagnostics: Grant/Research Support|Takeda: Advisor/Consultant|Takeda: Grant/Research Support|Trinity Biotech, LLC: Advisor/Consultant|Valneva: Advisor/Consultant|Valneva: Grant/Research Support **Daniel Bourque, MD**, Kephera Diagnostics: Grant/Research Support

